# Common Soft Tissue Tumors Involving the Hand with Histopathological Correlation

**DOI:** 10.25259/JCIS-6-2019

**Published:** 2019-05-24

**Authors:** Pankaj Nepal, Swachchhanda Songmen, Saeed Intakhab Alam, Darshan Gandhi, Neeta Ghimire, Vijayanadh Ojili

**Affiliations:** 1 Department of Radiology, St. Vincent’s Medical Center, Bridgeport, CT, USA; 2 Department of Clinical Imaging, Hamad Medical Corporation, Doha, Qatar; 3 Department of Pediatric Dentistry, Kathmandu University, Nepal; 4 Department of Radiology, University of Texas Health, San Antonio, TX, USA.

**Keywords:** Hand tumors, Tumor mimics, Magnetic resonance imaging

## Abstract

Soft tissue tumors involving the hand are common and most often benign. It is important to know the spectrum of soft tissue tumors of the hand and understand the typical as well as atypical imaging features are seen on different imaging modalities. The imaging features are largely determined by the tumor histopathology; thus, the basic idea about the tumor histopathology will always be useful. This article intends to focus on a comprehensive approach including demographics, clinical presentation, and imaging findings required to diagnose the tumor definitely or narrow the differentials. This article discusses common soft tissue tumor mimics of the hand as well, however, excludes the bone tumors for the sake of brevity.

## INTRODUCTION

Embryologically, mesodermal mesenchyme differentiates into different soft tissue structures such as muscle, tendon, synovial sheath, skin, blood vessels, and nerves. Thus, hand tumor may comprise these soft tissue structures. Although both hands represent a total of only 2% of total body surface area^[1]^ and a total of only 1.2% of total body weight, they account for 15% of all soft tissue tumors.^[2]^ Another interesting fact is that 95% of hand tumors without skin involvement are benign.^[3]^ Most of the hand tumors present early, given the superficial location and thus readily visible and palpable. Thus, for these reasons, most of the hand tumors have a good prognosis. Malignant soft tissue tumors of the hand are rare and represent only 2% of all hand lesions.^[4]^

## DISCUSSION

In the increasingly imaging-reliant clinical practice, it is not uncommon for the radiologists to evaluate soft tissue hand tumors. This article provides an overview of the classification of common soft tissue hand tumors and highlights the important, useful imaging features of these tumors [Table 1].

**Table 1:** Summary of imaging findings of common soft tissue tumors of hand and their mimics.

Hand pathology	Clinical characteristics	Characteristic imaging features
Hemangioma	30% congenital, present at birth. 70% involute by 7 years of age.	Intensely hyperintense on T2. Homogenous gadolinium enhancement. Involution phase: Fat replacement, reduced enhancement.
Vascular malformation Lipoma	Do not involute but grow proportionately to the host. Most common soft tissue tumor.	Slow versus high flow. Areas of signal voids, phleboliths Encapsulated fat containing tumor without thick septa or nodularity.
Fibroma of tendon sheath Pilomatricoma	Second-fifth decades, slow growing mass on flexor surface, mild tender. Child <2 years.	T1 and T2 hypointense, little or no enhancement. No blooming on GRE and absent of cortical erosions. US: Internal matrix calcification, peripheral hypoechoic rim.
Fibrolipomatous hamartoma Traumatic neuroma	Classic location along median nerve. Macrodactyly. History of traumatic injury.	“Coaxial cable,” “spaghetti appearance,” on MR. Classic imaging appearance. “Balloon on string” appearance, T2 hyperintense, occasional enhancement, non-specific.
Peripheral nerve sheath tumor Glomus tumor Tenosynovial giant cell tumor Synovial sarcoma	Location along the nerve, tinel sign +. 75% tumors involve hand along the fingertip, painful. Painless mass in 30–50 years adjacent to tendon sheath. 85% in hand. Mass near the joint in young age.	“Split fat sign,” target sign,” and “fascicular sign” on MRI. Avid contrast enhancement, T2 high signal. Low on T1 and T2 due to hemosiderin. Closest differential: PVNS. Calcifications in l/3^rd^. Well defined, benign appearing lobulated mass.
Squamous cell carcinoma Ganglion cyst	Elderly age group. Most common wrist region	Heterogeneous, hemorrhage, and necrosis, infiltrative, and lobulated margins. Purely cystic on US and MRI. May demonstrate smooth, thin rim enhancement.
Nodular fasciitis	Rapid growth, simulate malignancy clinically.	Most patients need biopsy due to non-specific imaging features.

MRI: Magnetic resonance imaging

## IMAGING MODALITIES AND TECHNIQUES

A plain radiograph is the initial imaging ordered to evaluate the soft tissue tumor. Although it is not able to demonstrate the soft tissue tumor itself in the majority of cases, important information about the tumors can be occasionally obtained, for example, phleboliths in hemangioma, distal phalanx remodeling in epidermoid inclusion cyst. Ultrasound is less commonly used, but useful modality for soft tissue hand tumors. Given the superficial location, high-frequency transducer can be used to obtain high-resolution images of the tumor characteristics, for example, cystic versus solid tumor component, entering, and exiting nerve in relation to nerve sheath tumor. Color Doppler can provide important information about the vascularity to aid in the diagnosis as well. In cross-sectional imaging, magnetic resonance imaging (MRI) is frequently performed to characterize the soft tissue hand tumor. MRI signals are determined by the tissue composition; thus, it is helpful for radiologist to have understanding of the tumor histopathology as well.^[5]^ By corroborating clinical history, lesion location, radiograph appearance, US finding, and MRI characteristics, it is possible to determine the diagnosis in subset of lesions, but other lesions will need workup with biopsy to exclude malignancy.^[6]^

## OUR INSTITUTIONAL MRI PROTOCOL

- T1 coronal and axial, T2 sagittal and axial,
- T2 Fat saturated/Short TI Inversion Recovery (STIR) sequences on axial,
- Postcontrast images on axial and coronal plane,
- Three dimensional images, gradient recalled echo (GRE) sequences for vascular lesions,
- FOV: 12–16 cm; slice thickness 1.5–4 mm; Matrix: 512 × 256.

## BENIGN TUMORS

### Vascular anomalies (hemangioma and vascular malformations)

The nomenclature of vascular anomalies in the literature is unclear often complicated and confusing. Based on clinical findings, cellular turnover and histology; vascular anomalies can be broadly classified into vascular neoplasms and vascular malformations. Hemangiomas are true neoplasms of children, while arteriovenous malformations consist of dysplastic vessels. Both are frequent in childhood and young age group.

### Hemangioma

**Figure 1:**
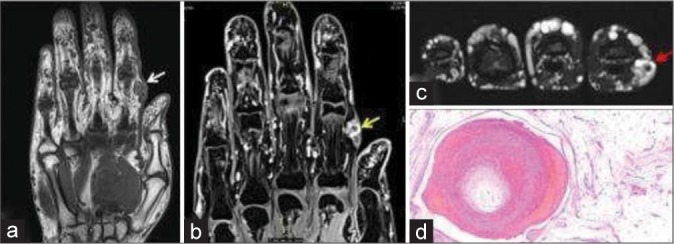
35-year-old woman with non-involuting congenital hemangioma who presented with painless swelling of index finger. (a) Non-contrast T1-weighted coronal magnetic resonance (MR) image shows a nodular hypointense lesion in subcutaneous and intramuscular plane of index finger (white arrow). (b) Fat saturated T1-weighted post-contrast MR image shows contrast enhancement (yellow arrow). (c) Axial T2-weighted image shows T2 flow void within the soft tissue indicating vascular etiology (red arrow). (d) Histopathology section showing lobules of mature adipocytes containing dilated thrombosed blood vessels with adjacent ragged blood vessels.

Up to 30% of hemangiomas are present at birth, and almost all are evident in a month.^[7]^ Initial rapid growth (growth of hemangioma disproportionately higher than the growth of the child, usually up to 1 year), then static phase (growth of hemangioma matches growth of the child, usually a few years), and ultimately slow involution is characteristic of hemangioma. About 50% involute by 5 years of age and about 70% involute by 7 years of age.^[8]^ Hemangioma does not recur. Apart from the vascular channels, the hemangioma may contain adipose tissue and phleboliths as well. On ultrasound, hemangioma appears moderately well defined and hypoechoic but can be heteroechoic due to internal adipose and calcium contents. Doppler may depict prominent vascular channels with the flow. Hemangioma is intensely hyperintense on T2 and hypointense on T1 unless have a hemorrhage or fatty replacement [Figure 1] Usually, they demonstrate homogenous enhancement after gadolinium administration. The phleboliths are seen as signal void on both T1- and T2-weighted images and may show blooming artifact on gradient echo images.

### Dysplastic vessels (vascular malformations)

**Figure 2:**
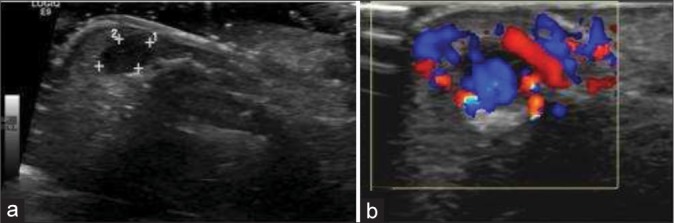
5-year-old boy with soft tissue arteriovenous (AV) malformation of hand who presented with slowly growing swelling of thumb. (a) Gray scale ultrasound image of the tuft of thumb shows a well-defined hypoechoic lesion (measured by calipers), which was misinterpreted as a well-defined cystic lesion. (b) Subsequent Doppler imaging of the lesion demonstrates significant color flow suggestive of AV malformation.

Unlike hemangioma, vascular malformations do not involute but grow proportionately to the host. The growth usually accelerates during puberty and pregnancy.^[9]^ Unlike hemangioma, vascular malformations are associated with bony destruction or skeletal hypertrophy. Imaging appearance depends on the type of vascular malformations – low flow versus high flow. Imaging may show size, location and internal characteristics, for example, phleboliths and vascular channels. Color Doppler is useful to see the internal vascularity including the arterial flow in case of high flow types [Figure 2]. MRI shows T1 hypointense and T2 hyperintense signal, signal voids (reflecting areas of high flow), and susceptibility artifacts (areas of thrombosis).^[10]^ Vascular malformations can be classified as either low-flow or high-flow lesions with dynamic contrast-enhanced magnetic resonance (MR) angiography.

### Lipoma

Lipoma is the most common soft tissue tumor. The most common location is subcutaneous but can be subfascial or intraosseous as well. The classic lipoma is composed entirely of fat, without areas of nodularity or thick septations. However, a significant proportion of lipomas may contain non-adipose components due to fat necrosis, calcification, fibrosis, inflammation, and myxoid degeneration. A study by Kransdorf *et al.* showed 31% (11 of 35) lipoma had non-adipose components.^[11]^

**Figure 3:**
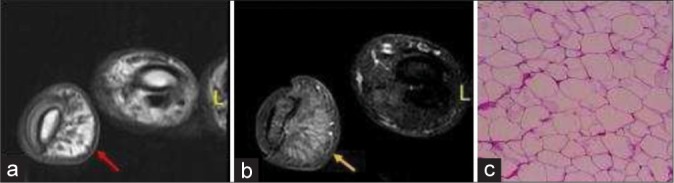
A 17-year-old female with lipoma of the finger presented with slowly progressive soft tissue swelling for years. (a) Non-contrast axial T1-weighted magnetic resonance image of the little finger showing fat and fibrous septa on volar surface (red arrow). (b) Axial fat saturated T2 weighted image demonstrates fat suppression (yellow arrow). (c) Histopathology confirmed the diagnosis revealing fat globules.

Lipoma variants, such as angiolipoma and myolipoma, also have non-adipose components. Clinically lipomas present as mobile,painless lump of soft tissue. Rarely they can present with symptoms of nerve compression if located at specific locations such as carpal tunnel, deep palmar surface and Guyon’s canal.^[3]^ On ultrasound, lipoma characteristically appears as an echogenic (isoechoic to fat and echogenic to muscle) well-defined lesion with echogenic capsule and fine internal echogenic striations parallel to the long axis of tumor.^[12]^ Internal vascularity is typically absent but might be seen in angiolipoma. On MRI, lipoma shows a similar signal to fat on all sequences, except areas of non-adipose components [Figure 3]. Although rare, malignant degeneration is possible when lipoma is painful and grows rapidly, along with features of tissue invasion on imaging.^[13]^ Important differentials are well-differentiated liposarcoma when the non-adipose component is seen. The features that favor liposarcoma over lipoma include old age, large size (>10 cm), thick septations (>2mm), nodular, or mass such as shape of the nonadipose component and fat content <75%.^[10]^

## FIBROMA OF TENDON SHEATH (FTS)

**Figure 4:**
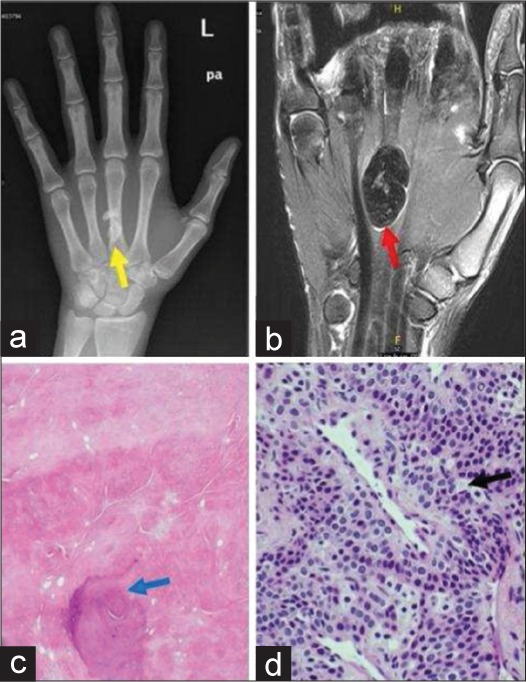
A 41-year-old male with fibroma of tendon sheath who presented with firm swelling on palm. (a) Plain radiograph of left hand shows a dense calcified lesion in metacarpal region (yellow arrow). (b) Non-contrast coronal T2-weighted magnetic resonance image of hand demonstrates profound T2 hypointense soft tissue mass along the palmar surface, deep to flexor digitorium tendons (red arrow). (c) Histopathology reveals bluish areas of calcification showing border between collagen fibers (blue arrow) (d) well-circumscribed nodules of dense fibrous tissue (black arrow) at ×40 magnification.

It is a benign soft tissue mass adjacent to the tendon sheath that occurs due to reactive fibrosis in adults between second and fifth decades. Histologically, the mass is composed of tightly packed spindle cells surrounded by collagen fibers. Approximately 82% of FTS occur in the hand and wrist region. The patient often presents with slow-growing mass with mild tenderness on examination. On the US, it appears homogenously hypoechoic and most commonly seen on flexor surface often in relation to an annular pulley.^[14]^ MR reveals a focal nodular mass adjacent to a tendon sheath with heterogeneous low to intermediate signal on all pulse sequences [Figure 4]. MR signal intensity on T2 sequence and contrast enhancement can be variable depending on cellular and fibrous component.^[15]^ It is most often confused with localized type tenosynovial giant cell tumor (TSGCT) both at clinical examination and even at gross pathology. TSGCT, generally, show susceptibility artifacts on GRE images and adjacent cortical erosion on a plain radiograph; both features are absent in FTS. Another differential is nodular fasciitis; however, nodular fasciitis is usually subcutaneous, but not associated with the tendon sheath.

## PILOMATRICOMA

**Figure 5:**
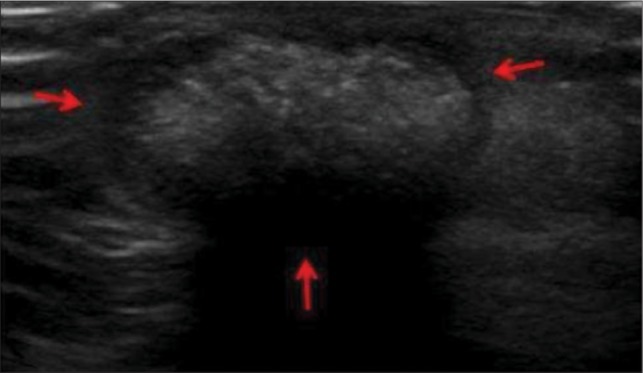
A 13-year-old boy with pilomatricoma who presented with dorsum of hand swelling. (a) Gray scale ultrasound image reveals superficial solid mass with typical hypoechoic rim (red arrows) and internal echogenic calcifications.

It is a benign tumor arising from the cortex of a hair follicle in the subcutaneous plane. It is common in the pediatric age group, typically <2 years but can occur at any age. The most common location is the head and neck region but can involve extremities. In ultrasound, it appears usually well defined, oval and hypoechoic relative to subcutaneous fat. It contains internal matrix calcification, with the most common pattern being scattered dot pattern [Figure 5].^[16]^ It has a hypoechoic peripheral rim, which represents a connective tissue capsule. It shows posterior acoustic shadowing as well. Intermediate internal vascularity can be seen in 50%.

An epidermoid cyst is a differential, but to the contrary, epidermoid shows internal anechoic cysts, linear hyperechoic strands, posterior acoustic enhancement, laminated concentric ring “target” pattern, pseudotestis appearance,^[17]^ and absence of internal vascularity.

## FIBROLIPOMATOUS HAMARTOMA (OF MEDIAN NERVE)

**Figure 6:**
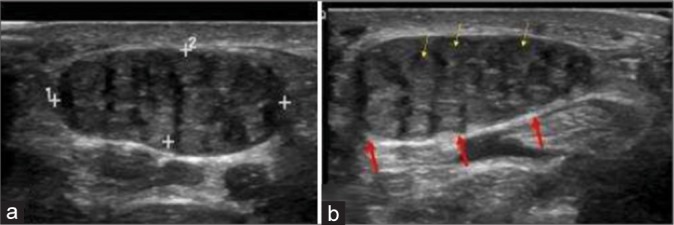
A 10-year-old girl with bilateral fibrolipomatous hamartomas of median nerve who presented with swelling around bilateral wrist and distal forearm. (a, b) Gray scale ultrasound images of proximal volar aspect of bilateral hands showing characteristic coaxial encasement of median nerves by solid echogenic heterogeneous substratum (red arrows). Note thin yellow arrows highlighting a few nerve fascicles on the background of fibrofatty replacement.

It is a benign neoplasm of a nerve, resulting from anomalous growth of fibroadipose tissue of the nerve sheath. By far, the most common location is the median nerve. It is usually congenital, thus seen at birth or infancy, but may be seen to the third decade.^[18]^ Dense fibrofatty tissues infiltrate the endoneurium, perineurium, and epineurium but nerve fascicles themselves remain intact. Macrodactyly is seen in 2/3^rd^ cases. Involvement of median nerve frequently causes carpal tunnel syndrome. The accepted treatment is carpal tunnel release by surgically excising the flexor retinaculum. Ultrasound shows cable-like appearance with alternating hyperechoic (due to fibrofatty tissue) and hypoechoic (neural tissue) bands. T1-weighted MRI image shows typical “coaxial cable” on the axial plane and “spaghetti appearance” in the sagittal plane due to alternating intermediate neural tissue and hyperintense fatty tissue [Figure 6].^[19]^

The differential is an intraneural lipoma, which is an encapsulated tumor that displaces the neural tissues eccentrically but does not infiltrate. The distinction is important since intraneural lipoma can be excised surgically given its non-infiltrative nature, but fibrolipomatous hamartoma cannot be excised surgically given its infiltrative nature. The age at presentation is different as well since intraneural lipoma occurs in the fourth or fifth decades.

## TRAUMATIC NEUROMA OF THE HAND

**Figure 7:**
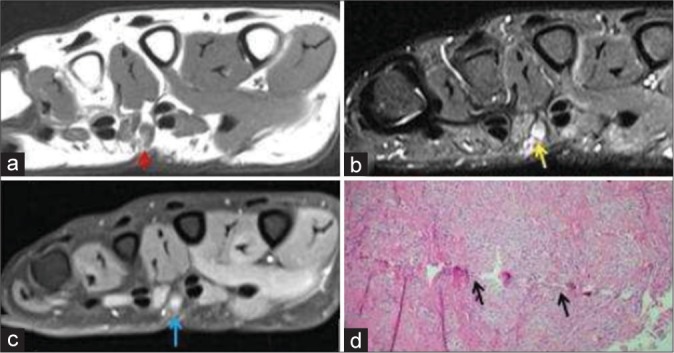
A 50-year-old male with traumatic neuroma presented with palmar swelling around scar site. (a) Non-contrast T1-weighted axial image shows a T1 hypointense small nodular lesion in palmar aspect of hand (red arrow). (b) The lesion is hyperintense on axial fat saturated T2-weighted image (yellow arrow). (c) It shows post-contrast enhancement (blue arrow). (d) Histopathology shows axons, Schwann cells, perineurial fibroblasts with scar tissue, thus confirming the diagnosis with a history of prior penetrating soft tissue injury.

Traumatic neuroma is the non-neoplastic proliferation of the injured or transected (completely or partially) proximal segment of the nerve. Traumatic neuromas are of two types: (a) Spindle neuroma: Swelling around an intact nerve after chronic irritation and (b) stump neuroma: Swelling at the end of a partially or completely transected nerve. They arise about 1–12 months post nerve injury. The characteristic symptom is intense neuralgia; Tinel’s sign is positive. A mass might be palpable if large enough. A neuroma is more common in lower limb and head/neck region, rather than the upper limb.^[20]^ Histology reveals a nonencapsulated disorganized collection of axons, Schwann cells, endoneurial/perineurial cells, and fibroblasts in scar tissue.^[21]^ On the US, it is seen as fusiform hypoechoic nodule in ultrasound, with entering/exiting nerve evident only if the neuroma is big enough. On MRI, it is seen as a T2 hyperintense nerve terminating in a baseball-shaped mass resembling a balloon on a string or a green onion appearance [Figure 7]. Traumatic neuroma can be distinguished from neurogenic tumors by the presence of surrounding scarring and lack of a split fat or target sign. Traumatic neuroma can occasionally enhance and thus nonspecific on imaging.

## PERIPHERAL NERVE SHEATH TUMORS (PNSTS) OF THE HAND

**Figure 8:**
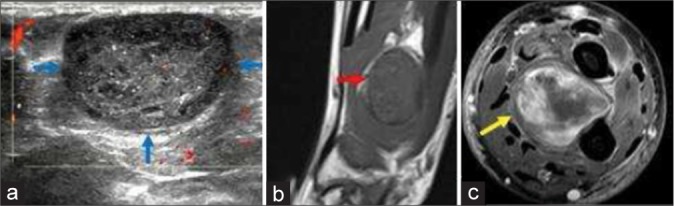
A 45-year-old woman with schwannoma who presented with swelling around wrist region. (a) Color Doppler ultrasound image showing a well-defined oval hypoechoic mass with smooth outline and hypoechoic rim. Note the trace internal vascularity within the mass (b) Non-contrast T1-weighted sagittal. Magnetic resonance images (MRI) image of the same patient showing well-defined hypointense mass with split-fat sign (red arrow). (c) Fat saturated axial T1-weighted contrast-enhanced MRI image shows heterogeneous enhancement of the mass (yellow arrow) with central necrosis.

Peripheral nerve sheath tumors of hand comprise schwannoma and neurofibroma. Schwannoma is a benign neoplasm of Schwann cells. Thus, it is often seen along the course of the nerve. It commonly presents in middle-aged adults without symptoms or with pain or paresthesia. A positive Tinel’s sign can be elicited, i.e., percussion to the mass will result in tingling and pain on the nerve distribution.^[22]^ It is well encapsulated and does not intertwine with the nerve fascicles; thus, complete surgical excision is easy without injuring the corresponding nerve (unlike neurofibroma, which is intertwined with the nerve fascicles, thus complete surgical excision of the tumor is not possible without sacrificing the nerve).^[22]^ The US shows well defined hypoechoic mass, with cystic degeneration and with long axis along the direction of the nerve. Color Doppler shows internal vascularity. MR shows T1 low-to-intermediate signal and T2 high signal with post-contrast homogeneous avid enhancement. The “split-fat sign” is the peripheral rim of perineural fat compressed by the tumor [Figure 8]. The “fascicular sign” is central small ring-like structures representing nerve fibers. The “target sign” is the central low signal of fibrocollagenous tissue with surrounding high signal of myxomatous on T2-weighted sequence.^[23]^ These signs are very useful to make the diagnosis of a nerve sheath tumor. Differentiation from neurofibroma is usually not possible with imaging. Cystic schwannoma might simulate a ganglion – entry and exit nerve roots can be seen with cystic schwannoma, which itself is a rare entity. On MR, it appears usually T1 hypointense (can be variable depending on the protein content) and T2 hyperintense. On post-contrast images, enhancement of the thin walls and septations is noted.^[21]^

## INTERMEDIATE TUMORS

### Glomus tumor of hand

**Figure 9:**
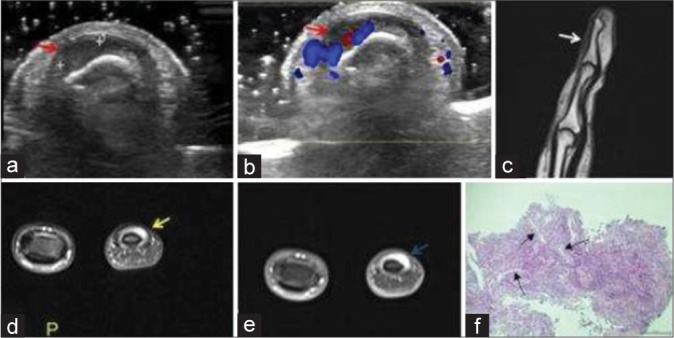
A 35-year-old female with glomus tumor who presented with painful subungual swelling. (a) Gray scale ultrasound image showing a well-defined hypoechoic mass in the subungual region (red arrow) (b) color Doppler shows internal vascularity. (c) The lesion (white arrow) is hypointense on T1W image (d) and hyperintense on axial T2W image with underlying pressure erosion (yellow arrow) (e) post-contrast axial T1W image demonstrates marked contrast enhancement (blue arrow) (f) histopathology reveals branching vascular channels (black arrows) separated by stroma containing glomus cells in nests.

Glomus tumor is hamartoma of glomus body. Glomus body is a special type of arteriovenous anastomosis in the dermis for thermoregulation. Glomus bodies are highly concentrated in digits, palms, and soles; thus, reflecting the predominant distribution of glomus tumor in these areas. Up to 75% of glomus tumors occur in hand; up to 65% occur at fingertips, predominantly at subungual space.^[24]^ The patient presents at 3^rd^-^5th^ decades in life with fingertip intense pain, extreme tenderness to minimal pressure and temperature sensitivity. Non-specific deformity and discoloration of the overlying nail are present, which is seen in any nail bed tumor due to very tight subungual space. The US shows well-defined small hypoechoic nodule beneath the nail bed, with adjacent distal phalangeal bony erosion. Doppler shows prominent internal vascularity, which is an important finding. MRI shows T1 low-to-intermediate, T2 high signal, and post-contrast homogenous intense enhancement. The avid enhancement is explained by the prominent vascular spaces interspersed with the stroma containing glomus cells [Figure 9]. The role of MRI is to diagnose the glomus tumor, to depict if multiple lesions are present and to properly localize the lesion preoperatively because the lesion is often too small to easily find during surgery.^[25]^

Differentials include hemangioma and epidermal inclusion cyst. Imaging features of hemangioma are similar, however presence of a fat component, phlebolith, calcification, and flow voids favor hemangioma over glomus tumor.^[26]^ Moreover, extreme pain and tenderness in glomus tumor also differentiates it from other differentials. Epidermal inclusion cyst can be differentiated from glomus tumor by the lack of internal vascularity but the only the presence of peripheral rim enhancement.^[27]^

### TSGCT

**Figure 10:**
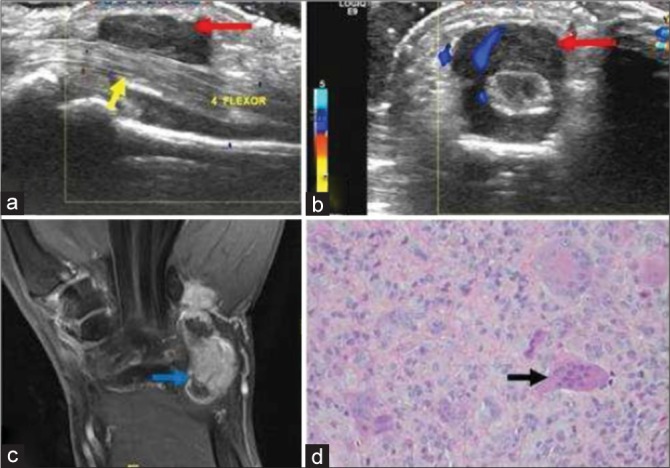
A 40-year-old male with giant cell tumor of tendon sheath who presented with progressive swelling of palmar surface of the hand. (a) Gray scale sagittal ultrasound image of the proximal hand demonstrates a well-defined hypoechoic soft tissue mass (red arrow) encasing the 4^th^ flexor muscle tendon of the hand (yellow arrow). (b) Color Doppler shows internal vascularity within the mass (red arrow). (c) Post-contrast fat saturated T1-weighted coronal image in another patient with the same diagnosis showing nodular heterogeneous enhancement (blue arrow). (d) Histopathology reveals mononuclear cells and osteoclast-like giant cells (black arrow).

Latest the WHO classification (2013) has replaced the term giant cell tumor of tendon sheath (GCTTS), pigmented villonodular tumor of the tendon sheath (PVNSTS), or extra-articular PVNSTS with TSGCT. It presents as a lobulated painless soft tissue mass immediately adjacent to tendon sheath. Typical age group is 30–50 years. It is almost always solitary; multifocal involvement is extremely rare with only five cases reported.^[28]^ The common locations are the hands (85% in fingers) or feet.^[29]^ Plain radiograph can show cortical erosions on the underlying bone ~ 20%. Periosteal reaction and calcification are uncommon. Ultrasound shows homogenous hypoechoic lesion with internal vascularity. With dynamic maneuver, it does not move with flexion or extension of the adjacent tendon. On MR, it shows T1 low- and T2 low-to-intermediate signal [Figure 10]. It shows intense contrast enhancement as well. The closest differential both radiological and histopathological is pigmented villonodular synovitis (PVNS); however, PVNS involves large joints. TSGCT tends not to bleed or hemorrhage unlike PVNS, so we tend not to see blooming artifact on GRE. TSGCT grows outward from the tendon sheath, whereas PVNS grows inward from the synovium into the joint.^[30]^ FTS is a differential, which does not show cortical erosion and “blooming.” Gouty tophi also appear T1 and T2 low, but tophi are usually multiple and intra-articular instead.

### Malignant tumors

#### Synovial sarcoma of the hand

**Figure 11:**
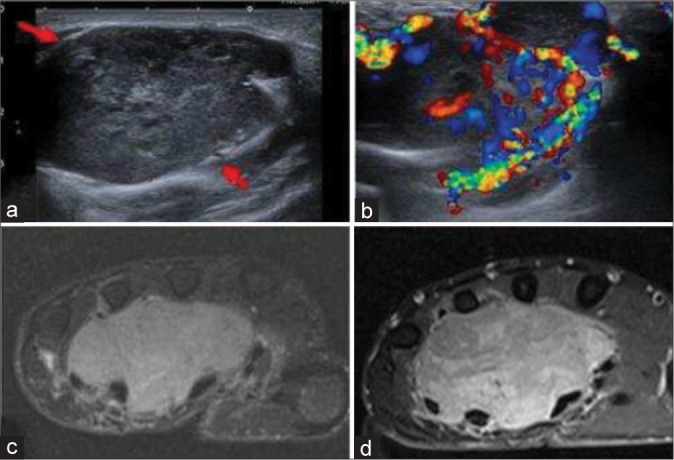
A 30-year-old woman with synovial sarcoma of hand who presented with slow-growing mass on hand. (a) Gray scale ultrasound image shows a well-defined solid and heterogeneous lobulated mass in volar surface of the right hand (red arrows). (b) Color Doppler shows significant color flow. (c) Fat saturated axial T2-weighted image showing hyperintense lobulated and insinuating mass, (d) with heterogeneous enhancement on post-contrast fat saturated T1-weighted axial image.

Synovial sarcoma is a relatively common tumor, representing 5–10% of all soft tissue sarcoma.^[31,32]^ It is intermediate to high-grade malignancy with high metastatic potential. Irrespective of the nomenclature, neither does it arise from the synovium nor does it express synovial markers. It can occur at any age and in any location.^[33]^ The majority (80–95%) occurs in the extremities; lower extremity is most often affected around the knee, whereas 16%–25% occur in the upper limb. Soft tissue mass near but not in a joint in a patient 15–40 years of age particularly if calcification is present, synovial sarcoma should be considered a possibility. Nonspecific calcification is seen in 1/3^rd^ of cases. It is frequently mistaken as benign pathology due to small size (<5 cm), slow indolent growth (average time to diagnosis 2–4 years, nonaggressive bony erosion), calcification, and well-defined lobulated outline. Imaging is not pathognomonic, but suggestive of the diagnosis. The US shows benign appearing hypoechoic, complex lesion with internal vascularity in solid component. Computed tomography is useful to show calcification and bone involvement. MRI shows multilobulated mass with marked heterogeneity with hemorrhage, fluid-fluid levels, and septa creating the “bowl of grapes” appearance. The marked heterogeneity aka “triple sign” on T2-weighted MR images is due to the result of the mixture of solid cellular elements, hemorrhage or necrosis, and calcified or fibrotic collagenized regions [Figures 11]. Predominantly cystic tumor shows areas of enhancing solid nodular areas, which can be targeted for image-guided biopsy for the definitive diagnosis.^[34]^

#### Squamous cell carcinoma

Squamous cell carcinoma occurs in the elderly age group. Diagnosis is usually suspected clinically and confirmed with a skin biopsy. Imaging features are variable, however heterogeneous signal due to fascial edema, hemorrhage and necrosis, irregular margins, and lobulation suggest the diagnosis of malignant etiology. MR imaging of the tumor is done for local staging, which shows infiltrative growth pattern, low to intermediate signal on T1W images and heterogeneously hyperintense on T2W images. Tumor shows heterogeneous and variable enhancement. An important diagnostic clue is irregular and infiltrative mass epicentered to cutaneous tissue.^[35]^

### Tumor mimics

#### Ganglion cyst

**Figure 12:**
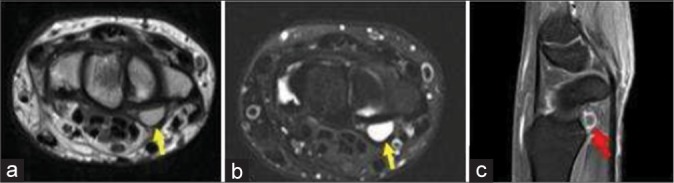
A 35-year-old man with ganglion cyst who presented with painless swelling over the proximal hand. (a) Non-contrast T1-weighted axial image showing a well-defined hyperintense lesion on the volar surface due to proteinaceous contents (yellow arrow). (b) Axial T2-weighted Magnetic resonance image at the level of wrist confirms a well-defined cystic lesion in volar surface adjacent to the flexure retinaculum (yellow arrow). (c) Fat saturated post-contrast sagittal T1 image shows rim enhancement of the cyst (red arrow).

Ganglion represents the most common cause of mass in the wrist area.^[36]^ Ganglion may form due to chronic irritation and degeneration of tendon sheath or may arise from a joint. Thus, it is located near the tendon sheath or joint. Most commonly it occurs in dorsum of the wrist from the scapholunate joint. It is most common in young adults and 3 times more common in females. The fluid is thick and mucoid. Since it is superficial and fluid filled, it can be readily imaged with ultrasound. Ultrasound can show hypoechoic fluid filled the smooth structure with thin septations; however, the connection with the joint of origin is difficult to image. It may be multilocular or variably echogenic depending on the protein content of the fluid. Color Doppler will show the absence of internal vascularity, which will differentiate from solid lesions as nerve sheath tumor and vascular anomalies. On MRI cyst are typically hyperintense on T1- weighted image due to proteinaceous contents and hyperintense on T2-weighted image [Figure 12]. MRI is invaluable in diagnosis of complex and deep cystic lesions that are otherwise difficult to evaluate with ultrasound.

### Nodular fasciitis of the hand

**Figure 13:**
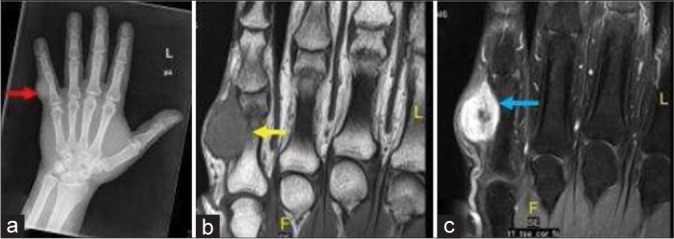
A 20-year-old male with nodular fasciitis of finger who presented with painful rapidly developing swelling in the proximal phalynx of ring finger. (a) Plain radiograph of the left hand demonstrates a well-defined soft tissue swelling in the proximal phalanx of the little finger (red arrow). Note absence of bony destruction or scalloping. (b) Coronal noncontrast T1-weighted image shows that the lesion is well defined and isointense to the muscle (yellow arrow). (c) The lesion demonstrates intense enhancement on fat saturated post-contrast T1-weighted image and is difficult to differentiate from sarcoma or giant cell tumor of tendon sheath.

Nodular fasciitis is a benign rapid reactive self-limiting proliferation of myofibroblasts leading to fibromatosis. It usually involves a subcutaneous plane, but can also affect fascia or muscle. 46% of cases are seen in the upper extremity, particularly the volar aspect of the forearm. Hand involvement is rare 0–2% and hand involvement also has a history of hand trauma in 38.5% of cases.^[37]^ The most commonly affected age group is 20–40 years. It is often misdiagnosed initially as sarcoma due to its rapid growth within month duration. Thus, incisional/excisional biopsy is required for the diagnosis; fine-needle aspiration is not adequate to make the diagnosis. Histopathologically, it contains abundant spindle-shaped myofibroblasts with typical mitotic figures, but without hyperchromasia and pleomorphism. Myxoid component predominates in the early lesion, but fibrous component predominates in the mature lesion.^[38]^ Imaging features are non-specific and depend on the internal content of fibrous, cellular, or myxoid components. The myxoid form is T1 isointense to hyperintense to skeletal muscle and T2 hyperintense to adipose tissue. Highly collagenous lesions have a hypointense signal on all MR images.^[39]^ Contrast enhancement is typically diffuse but may be peripheral in lesions with cystic changes or extracellular matrix [Figure 13].

The main differential is the sarcoma given the rapid growth and gross histopathology finding. Worrisome features suggestive of sarcoma over nodular fasciitis are age over 70 years, multiple lesions, lesion recurrence, perilesional tissue edema, or hemosiderin on MRI.^[40]^

### MRI as a problem solving tool

Benign tumors of hand mostly have well-defined margins and tend to show homogenous signal on T2W sequences. Hemangioma and schwannoma show homogenous contrast enhancement. Fatty lesions like lipoma have hyperintense signal on both T1 and T2W sequences. Fibrous lesions like FTS are hypointense on all MRI pulse sequences. On the other hand, malignant tumors have irregular infiltrative margins with lobulation. Malignant tumors show heterogeneous signal and heterogeneous enhancement due to fascial edema, hemorrhage, and necrosis. However, few exceptions exist, for example, malignant sarcoma may show well-defined margins and may insinuate the fascial and intermuscular plane like lipoma. On the other hand, desmoplastic fibroma has irregular infiltrative margins simulating malignancy. Intermediate lesions such as neurofibroma, GCTTS, and glomus tumor may show bone scalloping. Tumor mimics like nodular fasciitis simulate malignancy due to imaging features and rapid growth [Table 2].

**Table 2:**
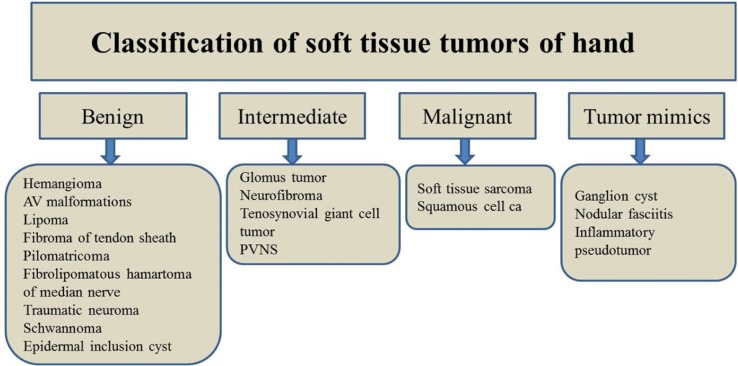
Classification of soft tissue tumors of hand and their mimics.

## CONCLUSION

Knowledge of typical and atypical imaging features of hand tumors is important to radiology practice. However, imaging features need to be interpreted in the background of patient demographics and clinical presentation as well. Although MRI is undoubtedly important imaging modality for soft tissue hand tumors, other initially ordered imaging modalities as radiographs and ultrasound can also add valuable information about the lesions. However, in many cases, ultimately patient will need a biopsy for a definitive diagnosis to rule out the malignant lesions.
